# *CXCL14* increase dendritic cell antigen presentation and promote asthma immune response

**DOI:** 10.1002/pdi3.2514

**Published:** 2025-01-05

**Authors:** Wenjie Wu, Fengxia Ding, Yan Li, Zhou Fu

**Affiliations:** ^1^ Department of Respiratory Medicine, Ministry of Education Key Laboratory of Child Development and Disorders National Clinical Research Center for Child Health and Disorders, China International Science and Technology Cooperation Base of Child Development and Critical Disorders, Chongqing Key Laboratory of Pediatrics, Chongqing Engineering Research Center of Stem Cell Therapy Children’s Hospital of Chongqing Medical University Chongqing China

**Keywords:** antigen presentation, asthma, C‐X‐C motif chemokine ligand 14, dendritic cells

## Abstract

Asthma is a chronic airway inflammatory disease characterized by reversible airflow limitation and airway hyperresponsiveness, which requires long‐term drug treatment and management. It is very important to study the etiology and pathogenesis of asthma for clinical asthma prevention and treatment. In this study, to understand the correlation between C‐X‐C motif chemokine ligand 14 (*CXCL14*) in bone marrow dendritic cells (BMDCs) and antigen presentation of asthma dendritic cells (DCs), an in vitro model of BMDCs was constructed for RNA sequencing (RNA‐seq). The changes of *CXCL14* in BMDCs after house dust mites (HDM) stimulation were evaluated. Finally, evaluated the inflammation of the lung tissue in mice, and the expression of costimulatory molecules on the DCs surface in the lung tissue was analyzed by flow cytometry. The results showed that *CXCL14* was upregulated in BMDCs after HDM stimulation, and its function was related to signal molecule interaction and the immune system. The expression of *CXCL14* was increased in the HDM‐induced allergic asthma model. Knockdown of *CXCL14* reduced the expression of costimulatory molecules CD86, CD80, and major histocompatibility complex II on the surface of DCs in the lung tissue of mice, induced immune tolerance, and reduced lung inflammatory cell infiltration and inflammatory factor levels, providing new ideas and theoretical basis for the clinical treatment of bronchial asthma.

## Graphical abstract

RNA‐seq showed that C‐X‐C motif chemokine ligand 14 (*CXCL14*) was upregulated in bone marrow dendritic cells after house dust mites (HDM) stimulation, and its function was related to signal molecule interaction and the immune system. The expression of *CXCL14* was increased in the HDM‐induced allergic asthma model. The reduction of *CXCL14* leads to the reduction of surface antigen‐presenting and co‐stimulatory molecules, induces immune tolerance, and alleviates lung inflammatory cell infiltration and cytokine levels.

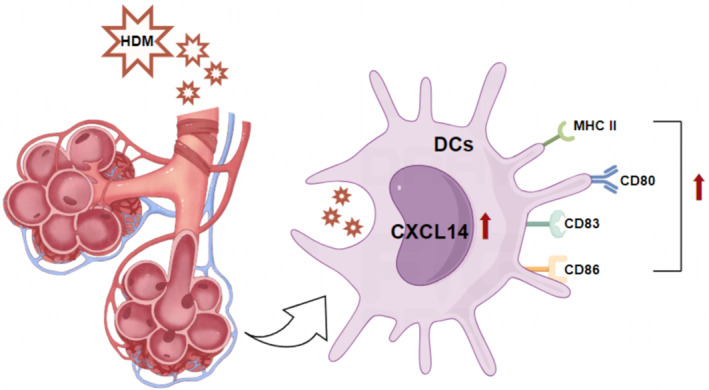

Asthma is a chronic airway inflammatory disease characterized by reversible airflow limitation and airway hyperresponsiveness. In recent years, the incidence of asthma continues to rise in many countries, especially in children and adolescents.^1^ Although modern medicine provides a variety of methods for the treatment of asthma, there are still a considerable number of patients who cannot completely control their symptoms. Therefore, it is very important to study the etiology and pathogenesis of asthma for clinical asthma prevention and treatment.

The pathogenesis of asthma is complex, and its inflammatory response is mainly mediated by two types of immune cells: adaptive immune response dominated by Th2 cells and innate immune response dominated by innate lymphocytes (ILCs).^2^ Dendritic cells (DCs), as potent antigen‐presenting cells, are recruited and activated by a variety of chemokines and cytokines secreted by airway epithelial cells after environmental stimulation, and promote their migration to the airway subcutaneous tissue.^3^ Activated DCs can capture and process exogenous antigens, and then present antigens to CD4 + T cells through major histocompatibility complex II (MHC II) molecules to initiate Th2 immune response.^4^

Chemokines are a class of small‐molecule proteins that can induce the directed migration of cells, playing a widespread role in immune surveillance and inflammatory responses within the body.^5^ *CXCL14* is an 8–14 kDa chemokine that primarily regulates cell migration and plays a crucial role in immune surveillance, inflammation, and cancer. *CXCL14* is widely expressed in various tissues, with particularly high levels in the breast and the kidney. Chemokines promote T cell–DC and T–B cell contact during antigen‐driven immune activation, suggesting that it may have significant functions within the immune system.^6^ Research has shown that *CXCL14* can promote the maturation and migration of DCs, enhancing their antigen‐presenting capability, thereby playing an important regulatory role in immune responses.^7^

Under basal conditions, monocyte‐derived immature DCs secrete low concentrations of *CXCL14*.^8^ Leukocytes expressing *CXCL14* can be chemotactically attracted by it, indicating that *CXCL14* has an immune surveillance function.^9^ By modulating the antigen presentation levels on the surface of DCs, asthma phenotypes can be alleviated. Investigating the role and mechanisms by which *CXCL14* mediates the regulation of bronchial asthma immune tolerance through DCs costimulatory molecules may provide new insights and theoretical foundations for the clinical treatment of bronchial asthma.

## 1 METHODS

### 1.1 Cell culture

Extract the femur and tibia from the mice (4–6 weeks), flush the bone marrow to obtain a cell suspension, and filter the bone marrow fluid through a mesh. Centrifuge the suspension at 1500 rpm at room temperature for 5 min. Prepare a complete culture medium containing fetal bovine serum and cytokines IL‐4 (final concentration of 10 ng/mL) and GM‐CSF (final concentration of 20 ng/mL). Mix the bone marrow cells thoroughly with the complete medium and then seed them for culture.

### 1.2 HDM stimulation of BMDCs

On day 8 of culture, collect the non‐adherent cells by gently pipetting. For the asthma group, add house dust mites (HDM) working solution (Greerlabs, Los Angeles, CA, USA) at a final concentration of 25 μg/ml. For the control group, add an equal volume of phosphate buffer saline (PBS) solution. After 24 h of stimulation, collect the bone marrow dendritic cells (BMDCs) for subsequent experiments.

### 1.3 Bioinformatics analysis

HDM‐stimulated BMDCs were established and sent to Shanghai Biotechnology Corporation for transcriptome sequencing. Differentially expressed genes (DEGs) between the two groups were identified based on the criteria of |log2FC| > 1 and *P* < 0.05. The “ggplot2″ *R* package was used to plot a volcano map and the “clusterProfiler” *R* package was employed for enrichment analysis. Gene Ontology (GO) and Kyoto Encyclopedia of Genes and Genomes (KEGG) enrichment analyses were conducted to identify related biological processes and pathways associated with these genes. GO functional enrichment and KEGG pathway enrichment analyses were performed using the clusterProfiler package in *R*, with a significant threshold of *P* < 0.05. Enrichment bubble plots were generated using the ggplot2 package.

### 1.4 Immunofluorescence protocol

Aspirate the existing medium from the confocal dish and fix the cells with 4% paraformaldehyde for 30 min. Wash with PBS three times, then permeabilize the cells with 0.5% Triton X‐100 (Solarbio, Beijing, China) for 20 min. Wash again with PBS three times. Block with 5% BSA (Solarbio, Beijing, China) at room temperature for 30 min. Add the *CXCL14* antibody (diluted in 5% BSA, 1:250, Abcam, Boston, MA, USA) and incubate overnight at 4°C. Wash with PBS three times, then add the FITC‐conjugated goat anti‐rabbit secondary antibody (diluted 1:250, Proteintech, Wuhan, China) and incubate in the dark at room temperature for 1 h. Stain with DAPI (Beyotime, Shanghai, China) in the dark at room temperature for 10 min. After mounting with anti‐fluorescence quencher (Beyotime, Shanghai, China), the images were captured by confocal microscope (Nikon, Tokyo, Japan).

### 1.5 Quantitative real‐time PCR (qRT‐PCR)

The expression levels of target genes were measured by qRT‐PCR. Total RNA was extracted using the RNA extraction kit (BioFLUX, Hangzhou, Zhejiang Province, China) following the provided instructions. Followed by reverse transcription using the AG11734 kit (Accurate Biology, Hunan, Hubei Province, China), and qPCR using 2×SYBR Green Pro‐Taq HS (Accurate Biology, Hunan, Hubei Province, China) according to the manufacturer's instructions. *β*‐actin was used as an internal reference for normalization. Data analysis was based on Ct values, and the relative gene expression compared to *β*‐actin was calculated using the 2^−△△Ct^ method.

The primer sequences are as follows (5′–3′).

**Table:** 

Genes	Primer sequences (5′–3′)
M‐*CXCL14*‐F	TACCCACACTGCGAGGAGAAGA
M‐*CXCL14*‐R	CGCTTCTCGTTCCAGGCATTGT
M‐*IL‐4*‐F	ATCATCGGCATTTTGAACGAGGTC
M‐*IL‐4*‐R	ACCTTGGAAGCCCTACAGACGA
M‐*IL‐5*‐F	GATGAGGCTTCCTGTCCCTACT
M‐*IL‐5*‐R	TGACAGGTTTTGGAATAGCATTTCC
M‐*IL‐10*‐F	CGGGAAGACAATAACTGCACCC
M‐*IL‐10*‐R	CGGTTAGCAGTATGTTGTCCAGC
M‐*IL‐13*‐F	TGAGCAACATCACACAAGACC
M‐*IL‐13*‐R	GGCCTTGCGGTTACAGAGG
M‐*β‐actin*‐F	GTGCTATGTTGCTCTAGACTTCG
M‐*β‐actin*‐R	ATGCCACAGGATTCCATACC

### 1.6 Western blot

Total protein extraction was performed according to the manufacturer's instructions (KeyGEN, Jiangsu, China). Protein concentration was determined using the BCA method (Beyotime, Shanghai, China). After preparing the electrophoresis gel, constant voltage electrophoresis was carried out. The polyvinylidene fluoride membrane was cut and activated with methanol. Following transfer at a constant current, the membrane was blocked with a rapid blocking solution (Beyotime, Shanghai, China) for 20 min and washed three times with Tris Buffered Saline with Tween‐20 (TBST). The *CXCL14* antibody was incubated overnight at 4°C followed by three TBST washes. The membrane was then incubated with the secondary antibody at room temperature for 1 h, washed with TBST three times, and developed. *β*‐*a* *ctin* was used as an internal control. Chemiluminescence detection was performed, and the resulting images were analyzed for grayscale intensity using ImageJ software.

### 1.7 Establishment of a mouse asthma model

Female C57BL/6 mice, 6–8 weeks old, were purchased from the Experimental Animal Center of Chongqing Medical University and housed in a specific pathogen‐free (SPF) facility. The mice were randomly divided with 5 mice in each group. On days 0 and 14, 40 μL of the HDM solution (20 μg) was administered intranasally to sensitize the mice, followed by intranasal administration of 40 μL HDM solution (20 μg) on days 21, 23, 25, 27, and 29 for challenge. Mice in the control group were sensitized and challenged with normal saline instead of HDM. All mice were sacrificed on day 31 for subsequent experimental analysis.

This study was approved by the Ethics Committee of the Children's Hospital of Chongqing Medical University. The animal experiments were conducted in accordance with the guidelines of the Institutional Animal Care and Use Committee (IACUC) and the Guide for the Care and Use of Laboratory Animals.

### 1.8 Construction of adeno‐associated virus‐transfected mice

AAV‐GFP and AAV‐sh*CXCL14* were constructed by viral therapy technologies. A total of 60 μL of solution containing 5 × 10^10^ AAV vector particles was intranasally administered to 4‐week‐old C57BL/6 mice, and the virus vector was diluted with normal saline. Two weeks later, the knockdown effect was verified, and the transfected mice were used to establish an HDM allergic asthma model.

### 1.9 BALF cell count

Mice were anesthetized via an intraperitoneal injection of 1% sodium pentobarbital. The pulmonary circulation was perfused with pre‐chilled PBS. The collected bronchoalveolar lavage fluid (BALF) was centrifuged to determine the total cell count. The cell pellet was used to prepare smears, which were air‐dried naturally. For eosinophil quantifcation, the remaining cells were stained with wright‐giemsa stain. The proportion of eosinophils was determined by counting a minimum of 500 cells, and the total eosinophil count was calculated.

### 1.10 Lung tissue H&E staining and scoring

Lung tissues were placed in 4% paraformaldehyde for fixation, followed by dehydration, embedding, and sectioning. The slides were deparaffinized in xylene and then rehydrated in a series of ethanol solutions with concentrations of 100%, 95%, 80%, and 75%. After staining with hematoxylin and eosin (H&E), the sections were dehydrated in an ethanol gradient, immersed in xylene, and finally mounted with neutral resin. Two independent observers examined the lung tissue sections under an optical microscope and documented the observed pathological changes. Inflammation around the trachea, bronchioles, blood vessels, and lung interstitium was evaluated, and each was scored from 1 to 3 according to the following criteria: (1) Peribronchial and peritracheal inflammation: The severity of inflammation was assessed by the thickness of the inflammatory cell infiltration around the airways. Several lumens were counted on each slide, and the average thickness was taken. 0 points, no infiltration; one point, infiltration less than 2 cell layers; two points, infiltration between three to five cell layers; and three points, infiltration greater than 5 cell layers. (2) Perivascular inflammation: 0 points, no infiltration; one point, infiltration less than 4 cell layers; two points, infiltration between five to seven cell layers; and three points, infiltration greater than 7 cell layers. (3) Interstitial inflammation: This refers to inflammatory infiltration in the alveolar septa. 0 points, no infiltration; one point, inflammatory infiltration without septal thickening; two points, obvious inflammatory infiltration with mild thickening; and three points, significant inflammatory infiltration with marked thickening.

### 1.11 Elisa

Mouse blood was collected from the eyeballs and centrifuged at 2500 rpm for 5 min at room temperature. The supernatant was collected and centrifuged again under the same conditions for 10 min to obtain the mouse serum. IgE levels were measured according to the instructions of the ELISA kit. After the reaction was stopped, the OD450 values were immediately measured, and data analysis was performed

### 1.12 Immunohistochemistry

After dewaxing and rehydrating the paraffin sections, antigen retrieval was performed followed by subsequent steps in accordance with the instructions provided in the kit (ZSGB‐BIO, Beijing, China). After the sections were mounted with neutral resin, images were captured. The mean option density was analyzed using ImageJ software.

### 1.13 Flow cytometry

Mouse lung tissue was digested with collagenase to prepare a single‐cell suspension, followed by blocking with rat serum at room temperature for 30 min. PerCP‐Cy5.5 isotype control and 2.5 μL anti‐CD11c‐PerCP‐Cy5.5 antibody (BD, Bergen, NJ, USA) were added and incubated at room temperature for 30 min. The cells were then washed twice with PBS at 4°C by centrifugation at 2500 rpm. The purity of BMDCs was assessed using flow cytometry.

### 1.14 Statistical analysis

Data were analyzed using GraphPad Prism 8.0 software, and results are expressed as mean ± SEM. Statistical comparisons were performed with analyzes of variance or two‐tailed Student’s t‐test with paired or unpaired wherever appropriate. A *p*‐value <0.05 was considered statistically significant.

## 2 RESULTS

### 2.1 Morphology and purity identification of BMDCs

**FIGURE 1:**
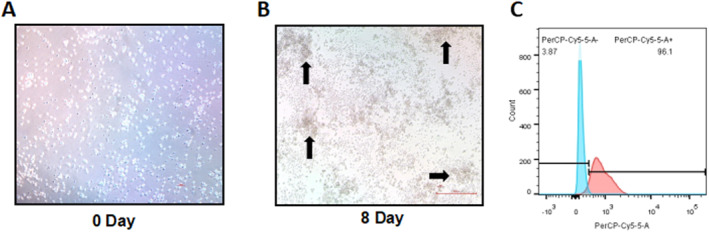
Morphological observation of BMDCs in mice of different days. Morphology of BMDCs on day 0 (A) and day 8 (B). Flow cytometry analysis of the percentage of CD11c + cells (C). BMDCs, bone marrow dendritic cells.

On the day of cell inoculation, cell morphology was observed under a light microscope (Figure 1A), with most cells suspended in the medium. On the eighth day, BMDCs colony formation was observed (Figure 1B), and typical BMDCs could be seen under a high‐power microscope. The proportion of CD11c + cells among BMDCs was greater than 85% (Figure 1C), indicating that the extracted cells met the required criteria and could be used for subsequent experimental studies.

### 2.2 RNA‐seq analysis

**FIGURE 2:**
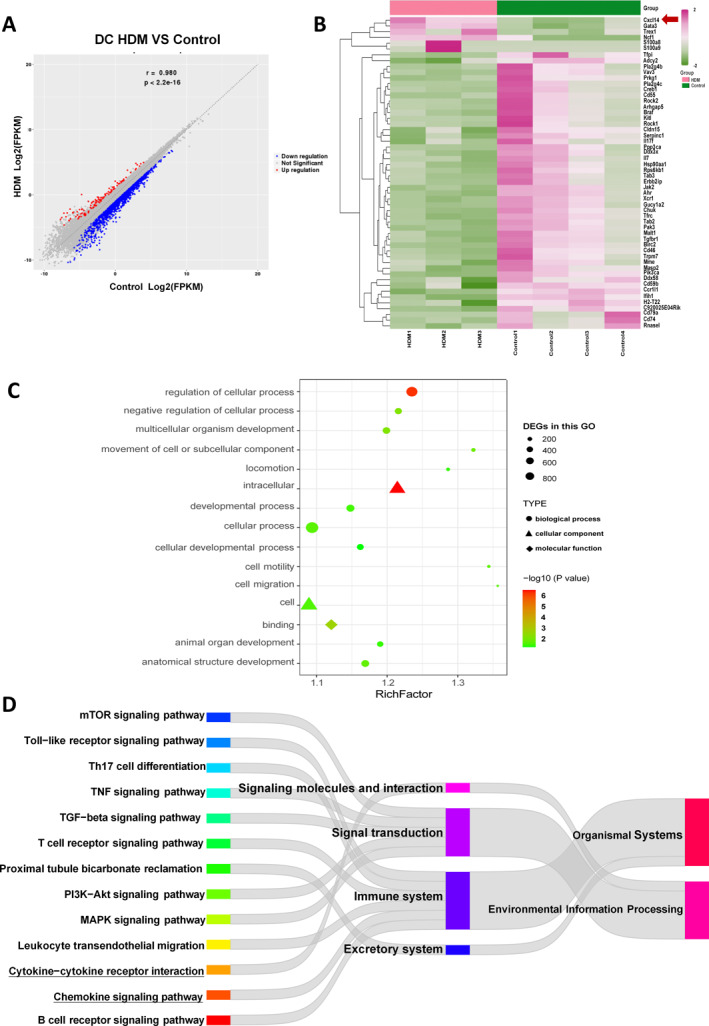
(A) DEGs volcano map, (B) heat map about DEGs, (C) the GO enrichment of the *CXCL14*, (D) the KEGG enrichment of the *CXCL14*. DEGs, differentially expressed genes.

A total of 1335 DEGs were identified through RNA‐seq analysis (Figure 2A). Among them, *CXCL14* was found to be upregulated in BMDCs following HDM stimulation (Figure 2B). GO enrichment analysis of *CXCL14* (Figure 2C) revealed its association with biological processes such as cell development, cell motility, and cell migration. Additionally, KEGG enrichment of *CXCL14* (Figure 2D) showed involvement in signal molecule interactions, including cytokine–cytokine receptor interaction, as well as pathways related to the immune system, such as the chemokine signaling pathway.

### 2.3 *CXCL14* expression increases after HDM stimulation in BMDCs

**FIGURE 3:**
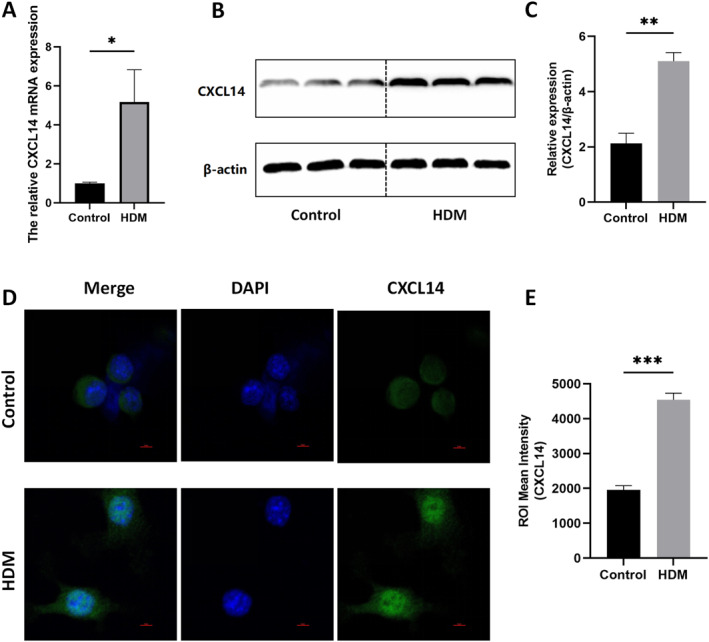
(A) *CXCL14* mRNA expression after HDM stimulation of BMDCs. (B) Representative western blots of *CXCL14* protein and (C) quantification of western blotting results (*n* = 3 per group). (D) Immunofluorescent staining for *CXCL14* and (E) ROI Mean Intensity. BMDCs, bone marrow dendritic cells; HDM, house dust mites.

To validate the changes in *CXCL14* expression, we performed qRT‐PCR (Figure 3A, *P < 0.05) and western blot (Figure 3B) analysis (Figure 3C, **P < 0.01). The results confirmed that *CXCL14* expression was significantly increased in the HDM‐stimulated group. Immunofluorescence (Figure 3D) assays further revealed that BMDCs transitioned from a round shape to a dendritic form after HDM stimulation, indicating maturation from an immature to a mature state, accompanied by an increase in *CXCL14* expression (Figure 3E, ***P < 0.001). These findings suggest that elevated *CXCL14* expression in the in vitro asthma BMDCs may be related to its role in immune mechanisms.

### 2.4 *CXCL14* expression in the lung tissue of asthma mice

**FIGURE 4:**
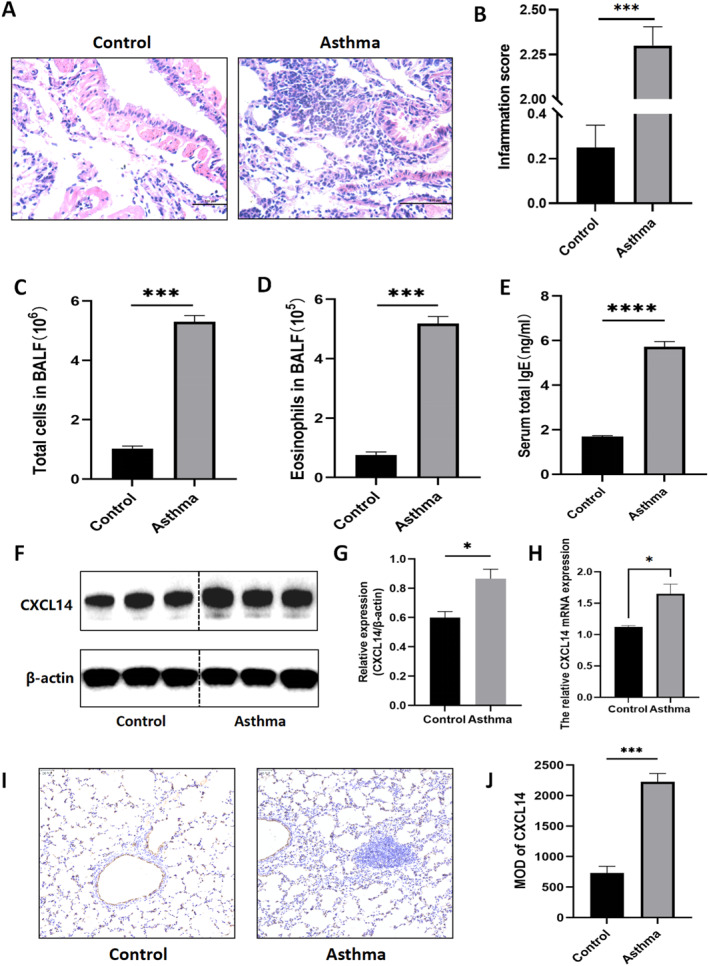
(A) H&E staining of lung sections and (B) inflammation score. (C) Total cells and (D) eosinophils in BALF. (E) ELISA for total IgE in mouse serum. (F) Representative western blots of *CXCL14* protein and (G) quantification. (H) *CXCL14* mRNA expression. (I) Immunohistochemical staining for *CXCL14* in the lung tissues and (J) MOD of *CXCL14*. BALF, bronchoalveolar lavage fluid; H&E, hematoxylin and eosin; MOD, mean option density.

H&E staining results showed significant infiltration of inflammatory cells around the bronchi in the asthma group, while no notable inflammatory cell infiltration was observed in the control group (Figure 4A). The inflammation score of the lung tissue in the asthma group was significantly higher than control group (Figure 4B, ***P < 0.001). Additionally, the total number of cells (Figure 4C, ***P < 0.001) and eosinophils (Figure 4D, ***P < 0.001) in BALF were elevated in the asthma group compared to the control mice. The IgE levels in the asthma group were also significantly higher than those in the control group (Figure 4E, ****P < 0.0001), indicating that the HDM‐stimulated mice were in an allergic state. Together, these results confirm the successful establishment of the asthma mouse, which is suitable for subsequent experiments.

Western blot (Figure 4F) and qRT‐PCR (Figure 4H) analyses demonstrated that *CXCL14* expression was significantly higher in the lung tissues of asthma mice compared to the control group. Quantification of band intensity using software indicated a statistically significant difference between the groups (Figure 4G, *P < 0.05). Immunohistochemistry results (Figure 4I, ***P < 0.001) further showed a pronounced increase in *CXCL14* expression in the asthma group. After quantification using ImageJ software, a statistically significant difference was observed in *CXCL14* protein expression between the two groups (Figure 4J, ***P < 0.001). These findings indicate that *CXCL14* expression is elevated in the lung tissue of asthma mice.

### 2.5 Validation of *CXCL14* knockdown in mouse lungs

**FIGURE 5:**
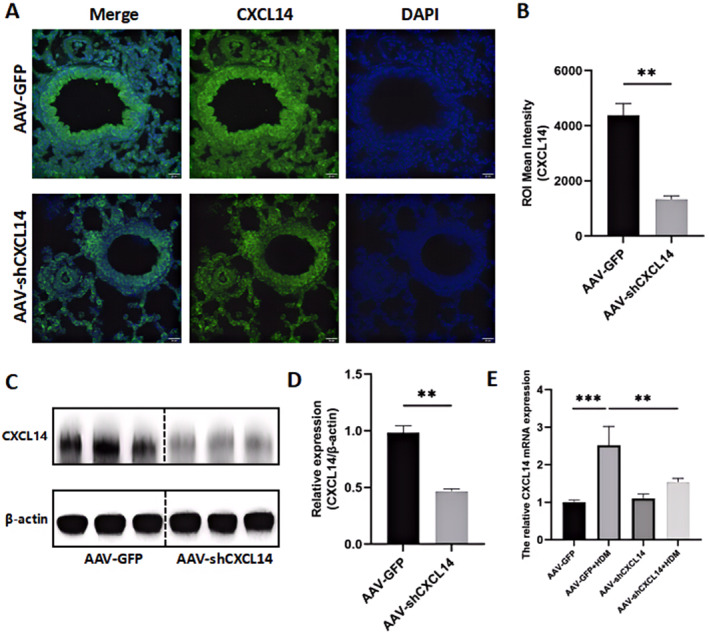
(A) Immunofluorescent staining for *CXCL14* and (B) ROI Mean Intensity. (C) Representative western blots of *CXCL14* protein and (D) gray value quantification. (E) *CXCL14* mRNA expression.

First, we used immunofluorescence to observe the lung tissue (Figure 5A), and a significant reduction in *CXCL14* fluorescence intensity was observed in the alveolar regions (Figure 5B, **P < 0.01), suggesting that *CXCL14* expressed by DCs may have been successfully knocked down. To further validate this, we extracted the lung tissue DCs and performed western blot analysis (Figure 5C), which showed that *CXCL14* protein expression in the AAV‐sh*CXCL14* group was significantly reduced compared to the AAV‐GFP group (Figure 5D, **P < 0.01). These results indicate that *CXCL14* on both the lung tissue and the surface of lung DCs was successfully knocked down.

Moreover, qRT‐PCR results (Figure 5E) showed that the mRNA levels of *CXCL14* were significantly elevated in the AAV‐GFP + HDM group compared to the AAV‐GFP group (***P < 0.001), whereas the AAV‐sh*CXCL14* + HDM group exhibited a marked decrease in *CXCL14* mRNA levels compared to the AAV‐GFP + HDM group (**P < 0.01).

### 2.6 *CXCL14* knockdown reduces antigen presentation of lung DCs

**FIGURE 6:**
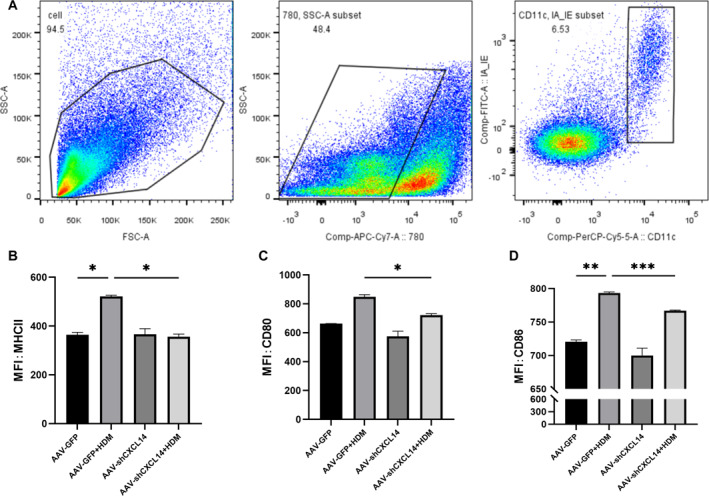
(A) The phenotype of DCs were assessed by flow cytometry. Representative histogram shows the expression of MHC II (B), CD80 (C), CD86 (D). DCs, dendritic cells; MHC II, major histocompatibility complex II.

MHC II, CD80, and CD86 are costimulatory molecules expressed on the surface of DCs. We used flow cytometry to assess the expression of these costimulatory molecules in lung tissue DCs (Figure 6A). The results showed that the expression of costimulatory molecules on DCs was elevated in the lung tissue of asthma mice. After *CXCL14* knockdown, the expression of MHC II (Figure 6B, *P < 0.05), CD80 (Figure 6C, *P < 0.05), and CD86 (Figure 6D, ***P < 0.001) was significantly reduced. This suggests that antigen presentation by DCs is decreased following *CXCL14* knockdown.

### 2.7 Knockdown of *CXCL14* reduced inflammation in the lung tissue of asthma mice

**FIGURE 7:**
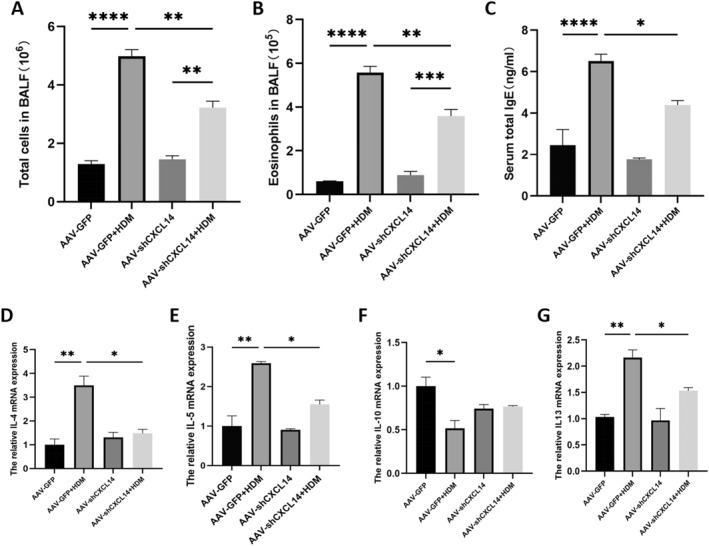
(A) Total cells and (B) eosinophils in BALF and (C) ELISA for total IgE in mouse serum. (D) *IL‐4*, (E) *IL‐5*, (F) *IL‐10*, and (G) *IL‐13* mRNA expression in the lung. BALF, bronchoalveolar lavage fluid.

Following *CXCL14* knockdown, both the total cells in BALF (Figure 7A, **P < 0.01) and the absolute eosinophils count (Figure 7B, **P < 0.01) were reduced compared to the AAV‐GFP group in asthma mice. ELISA results showed that serum total IgE levels in asthmatic mice were lower in the *CXCL14* knockdown group than in the AAV‐GFP group (Figure 7C, *P < 0.05).

As antigen‐presenting cells, DCs can trigger Th2 cells and innate immune cells to secrete cytokines. qRT‐PCR results revealed that inflammatory cytokines *IL‐4*, *IL‐5*, and *IL‐13* mRNA levels were significantly elevated in the lung tissues of asthmatic mice compared to the control group, while *IL‐10* levels were decreased. After *CXCL14* knockdown, the expression levels of *IL‐4* (Figure 7D, *P < 0.05), *IL‐5* (Figure 7E, *P < 0.05), and *IL‐13* (Figure 7G, *P < 0.05) mRNA were significantly reduced compared to the AAV‐GFP + HDM group. *IL‐10* (Figure 7F) mRNA levels were higher in the *CXCL14* knockdown group compared to the AAV‐GFP + HDM group, but the difference was not statistically significant.

## 3 CONCLUSIONS

Chemokines are a class of small proteins that regulate the migration, localization, and function of immune cells by binding to their corresponding receptors.^10^ In the pathological mechanism of asthma, chemokines exacerbate airway inflammation by influencing immune cell migration and activation. Specific chemokines, such as *CCL11*, *CCL5*, and *CXCL12*, have been shown to play critical roles in airway inflammation in asthma by promoting the migration and activation of eosinophils and DCs, thereby driving excessive inflammatory responses in the airways.^11^ Therefore, studying the regulatory roles of chemokines in asthma, particularly their impact on dendritic cell function, is essential for understanding asthma pathogenesis.

*CXCL14* is an atypical chemokine with complex functions and mechanisms of action. It is broadly expressed in various tissues and cell types, including epithelial cells, endothelial cells, and immune cells. Studies have shown that *CXCL14* plays an important role in the maturation and activation of DCs. By inducing the migration of DCs, *CXCL14* promotes their transfer to inflammatory sites or lymph nodes, increasing the expression of MHC II molecules and costimulatory molecules (such as CD80 and CD86) on DCs, thereby enhancing antigen presentation and T‐cell activation.^12^ Additionally, *CXCL14* has been found to contribute to immune tolerance, particularly in the regulation of DC‐mediated generation of regulatory T cells.^13^ This study found that *CXCL14* promotes the maturation and activity of DCs, and knocking down *CXCL14* can suppress excessive immune responses under certain conditions, which is crucial for balancing the immune response in asthma.

*CXCL14* not only promotes inflammatory responses by enhancing the chemotactic activity of DCs but may also influence DCs' role in immune tolerance by regulating their migration patterns. For example, under noninflammatory conditions, *CXCL14* may help maintain immune homeostasis by guiding DCs to migrate to lymph nodes and interact with regulatory T cells (Tregs), thereby reducing excessive inflammatory responses.^14^ This study found that *CXCL14* not only promotes DC migration to the airways but also enhances their antigen presentation capacity, further promoting the production of Th2‐type cytokines (such as *IL‐4*, *IL‐5*, and *IL‐13*), which facilitates eosinophil recruitment. The release of these proinflammatory cytokines leads to persistent airway inflammation, increasing airway hyperreactivity and worsening asthma symptoms.

Moreover, *CXCL14* regulates local immune responses in the airways during asthma by affecting DC maturation and activation. Under high inflammatory conditions, *CXCL14* induces DCs to express higher levels of MHC II molecules and costimulatory molecules, enabling them to more effectively activate Th2 cells and exacerbate allergic inflammatory responses. Therefore, *CXCL14* may play an important role in both acute exacerbations and the maintenance of chronic airway inflammation in asthma. By modulating the migration and maturation of DCs to lymph nodes, *CXCL14* also induces Treg expansion. Inhibiting *CXCL14* production may help suppress excessive airway remodeling and chronic inflammation in asthma patients, thereby slowing disease progression.

In conclusion, this study found that *CXCL14* expression is increased in asthmatic DCs, along with elevated levels of costimulatory molecules and antigen presentation.The reduction of *CXCL14* leads to the reduction of surface antigen‐presenting and costimulatory molecules, induces immune tolerance, and alleviates lung inflammatory cell infiltration and cytokine levels. Exploring the role and mechanism of *CXCL14* in DCs phenotype remodeling and bronchial asthma will contribute to a better understanding of asthma pathogenesis and provide essential targets for its prevention and treatment.

## AUTHOR CONTRIBUTIONS

Wenjie Wu and Fengxia Ding designed and performed the experiments, and wrote the manuscript. Yan Li designed the experiments, analyzed and interpreted the data. Zhou Fu contributed to the conception and design of the study, the revision of the manuscript, and the final approval of the version to be published.

## CONFLICT OF INTEREST STATEMENT

No financial or non‐financial benefits have been received or will be received from any party related directly or indirectly to the subject of this article.

## ETHICS STATEMENT

The study was approved by the Ethics Committee of Chongqing Medical University (20220804).

## Data Availability

The data that support the findings of this study are available from the corresponding author upon reasonable request.

## References

[1] Chen X , Zhou C‐W , Fu Y‐Y , et al. Global, regional, and national burden of chronic respiratory diseases and associated risk factors, 1990‐2019:Results from the Global Burden of Disease Study 2019. Front Med. 2023;10:1066804. 10.3389/fmed.2023.1066804 PMC10088372 37056726

[2] Busse WW , Lemanske RF . Asthma. N Engl J Med. 2001;344(5):350‐362. 11172168 10.1056/NEJM200102013440507

[3] Kool M , Willart MAM , van Nimwegen M , et al. An unexpected role for uric acid as an inducer of T helper 2 cell immunity to inhaled antigens and inflammatory mediator of allergic asthma. Immunity. 2011;34(4):527‐540. 21474346 10.1016/j.immuni.2011.03.015

[4] Hammad H , Lambrecht BN . Dendritic cells and epithelial cells: linking innate and adaptive immunity in asthma. Nat Rev Immunol. 2008;8(3):193‐204. 18301423 10.1038/nri2275

[5] Zlotnik A , Yoshie O . Chemokines: a new classification system and their role in immunity. Immunity. 2000;12(2):121‐127. 10714678 10.1016/s1074-7613(00)80165-x

[6] Moser B , Loetscher P . Lymphocyte traffic control by chemokines. Nat Immunol. 2001;2(2):123‐128. 11175804 10.1038/84219

[7] Ebert LM , Schaerli P , Moser B . Chemokine‐mediated control of T cell traffic in lymphoid and peripheral tissues. Mol Immunol. 2005;42(7):799‐809. 15829268 10.1016/j.molimm.2004.06.040

[8] Salogni L , Musso T , Bosisio D , et al. Activin A induces dendritic cell migration through the polarized release of CXC chemokine ligands 12 and 14. Blood. 2009;113(23):5848‐5856.19339694 10.1182/blood-2008-12-194597

[9] Sleeman MA , Fraser JK , Murison JG , et al. B cell‐ and monocyte‐activating chemokine (BMAC), a novel non‐ELR alpha‐chemokine. Int Immunol. 2000;12(5):677‐689. 10784614 10.1093/intimm/12.5.677

[10] Zlotnik A , Yoshie O . The chemokine superfamily revisited. Immunity. 2012;36(5):705‐716. 22633458 10.1016/j.immuni.2012.05.008 PMC3396424

[11] Humbles AA , Lloyd CM , McMillan SJ , et al. A critical role for eosinophils in allergic airways remodeling. Science. 2004;305(5691):1776‐1779. 15375268 10.1126/science.1100283

[12] Meuter S , Moser B . Constitutive expression of CXCL14 in healthy human and murine epithelial tissues. Cytokine. 2008;44(2):248‐255. 18809336 10.1016/j.cyto.2008.08.009

[13] Kurth I , Willimann K , Schaerli P , Hunziker T , Clark‐Lewis I , Moser B . Monocyte selectivity and tissue localization suggests a role for breast and kidney‐expressed chemokine (BRAK) in macrophage development. J Exp Med. 2001;194(6):855‐861. 11561000 10.1084/jem.194.6.855 PMC2195966

[14] Ebert LM , Schaerli P , Moser B . Chemokine‐mediated control of T cell traffic in lymphoid and peripheral tissues. Mol Immunol. 2005;42(7):799‐809. 15829268 10.1016/j.molimm.2004.06.040

